# Exploring the Perception and Experiences of Nursing Instructors With E-learning During the COVID-19 Pandemic in Saudi Arabia

**DOI:** 10.7759/cureus.42386

**Published:** 2023-07-24

**Authors:** Yahya Abdalla

**Affiliations:** 1 Nursing, Najran University, Najran, SAU

**Keywords:** community nursing, perception levels, covid-19 pandemic, perception, e-learning

## Abstract

Background/objective: Due to the coronavirus disease 2019 (COVID-19) pandemic's effect on the educational system, the integration of e-learning became essential. Consequently, it is necessary to explore nursing instructors' perceptions and experiences with e-learning. This study aims to explore the perception and experiences of nursing instructors in Saudi Arabia regarding e-learning.

Methods: A descriptive cross-sectional survey was conducted among nursing instructors at Saudi Arabian universities during the COVID-19 pandemic. They were invited to respond to an online questionnaire. The data collected were analyzed utilizing SPSS version 22 (IBM Corp., Armonk, NY), employing both descriptive statistics to summarize the responses of the participants and inferential statistics to identify significant differences in perception and experience levels based on demographic variables.

Results: The results demonstrated that nursing instructors in Saudi universities had a high perception of e-learning, with an average rating of 4.55/5. Instructors aged 41-50 years, of Indian or Saudi nationality, and holding a doctoral degree exhibited significantly higher levels of technological and computer experience compared to others. Moreover, instructors in these categories also had significantly higher levels of perception toward e-learning, with an overall average rating of 4.56/5.

Conclusions: The study concluded that nursing instructors at Saudi universities possessed a high level of experience in technology and computer usage, and they exhibited a favorable perception toward e-learning during the COVID-19 pandemic. Instructors aged 41-50 years, of Indian or Saudi nationality, and with a doctoral qualification demonstrated higher levels of experience in technology and computer usage, as well as a more positive perception of e-learning during the pandemic.

## Introduction

During the previous decades, there was a technological revolution that digitally improved learning tools and techniques, thus, e-learning become a vital part of several curricula and is gaining widespread acceptance as an appropriate alternative to traditional classrooms [1]. Furthermore, the international recommendations indicated that a faculty widespread practice of e-learning should be an essential element in the strategic progress of medical programs [2], as e-learning offers the advantages of lower cost, more access, and shared facilities and provides a facilitated learning process.

The e-learning approach provides a tool for the transition from traditional learning to a modern student-based approach [3]. On the other hand, there are certain issues faced by students as they do not have regular classes where they can go and discuss conceptual issues of different subjects besides lacking social and emotional connectivity [4]. The Blackboard platform (Blackboard Inc., Reston, VA) holds substantial worldwide importance as an educational system, predominantly in Saudi universities, where it plays a crucial role in facilitating online learning during the pandemic. Its flexibility helps instructors construct course content and enables students to access the content, submit assignments, and interact with peers and instructors. The platform's integration has been instrumental in adapting to the challenges posed by the coronavirus disease 2019 (COVID-19) pandemic, ensuring an uninterrupted educational environment [5].

End-user satisfaction refers to the degree to which users perceive that the e-learning approach meets their requirements. Understanding users' attitudes toward the approach is crucial in designing suitable educational environments [6,7]. Nevertheless, it is not easy to identify the factors influencing satisfaction [8]. Despite that, we still need to examine user satisfaction to obtain different alternatives for education [9].

Previous studies have approved that many instructors reflected satisfaction in using e-learning in teaching [10-12]. On the contrary, some instructors articulate concerns regarding the anticipated lack of interaction among students during online learning. They are afraid that the absence of in-person interactions may hinder participation, reduce chances for feedback, and limit teamwork development [13]. Furthermore, they perceive e-learning as time-consuming and labor-intensive [14]. Despite the rapid improvement of e-learning in the educational system globally, limited studies have been carried out on the perceptions and experiences of nursing educators specifically in the context of Saudi Arabia. This study was conducted to fill this gap in this field by exploring nursing instructors' perspectives on emergent e-learning and understanding its impact on teaching practices, thereby contributing valuable insights to educational policymakers and practitioners. Thus, this study aims to assess nursing instructors’ perceptions and experiences toward emergent e-learning at universities in Saudi Arabia.

## Materials and methods

Study design

This descriptive cross-sectional study aims to evaluate the perception and experiences of nursing instructors in nursing faculties in Saudi Arabia during the emergency shift toward e-learning.

Study participants

The study sample consisted of all online instructors who taught at nursing faculties in Saudi universities during the COVID-19 pandemic. The study is limited to those who are currently teaching at Saudi Universities from different nationalities.

Sampling and sample size

A random probability sampling technique was employed to select the 14 nursing colleges or departments at Saudi universities. The link for the online questionnaire was distributed to the selected faculties and the responses were 113, while 13 were excluded due to incomplete or inaccurate data included. The included sample size was 100 nursing instructors from the 14 selected Saudi universities. The questionnaire was sent to participants through social media (WhatsApp, emails). Thus, the estimated response rate for the online questionnaire is approximately 75.3%, indicating a reasonably good level of engagement from the nursing instructors in the study.

Data collection

All nursing instructors in Saudi universities were invited to participate in the study via an online link. Information about the survey and a Google Forms (Google, Mountain View, CA) questionnaire link was provided. All responses were anonymously and confidentially kept.

Data collection tools

A questionnaire designed by Liaw et al. (2007) to measure instructors' perceptions and experiences of e-learning was adopted and tested. The instrument employed a five-point Likert response scale, with the following numerical values assigned: strongly agree = 5, agree = 4, uncertain = 3, disagree = 2, and strongly disagree = 1. The questionnaire was administered in English. The reliability of this scale according to Cronbach’s alpha scale was 0.95. Furthermore, the correlation coefficient indicated a significant relation, and therefore it was acceptable for use in this study [15].

The questionnaire consisted of three major parts: (a) demographic information; (b) computer and internet experience; and (c) instructor’s satisfaction with e-learning. The demographic component, such as gender, and the field of teaching were included, and participants were also asked to express their experience with the internet and technical skills, and the last part included the satisfaction level of instructors.

Data analysis

The analysis of the study data was performed using SPSS version 22 (IBM Corp., Armonk, NY). Descriptive analysis for frequencies and percentages was applied, and differences were tested using means and standard deviations (SD) of the variables by independent t-test (for binary variables) and one-way ANOVA test (more than two options). A p-value of less than 0.05 was considered statistically significant.

## Results

Table 1 shows the demographic characteristics of participants in this study. Their ages were between 20 and 50 years. The specialties of nursing instructors were as follows: medical-surgical nursing (45%), community health nursing (18%), obstetrical & gynecological nursing (17%), pediatric nursing (12%), and critical care nursing (8%). The participants were Saudi (30%), Egyptian (26%), Sudanese (22%), Jordanian (8%), Filipino (6%), and Indian or Yemeni (4% each). The qualification of the participants included a doctorate (95%), master's (4%), or bachelor's degree (1%). Females represent 66% while males represent 34% of the participants. They worked at the universities for eight to 11 years (55%), four to seven years (32%), less than three years (8%), and 5% worked for more than 11 years.

**Table 1:** Demographic characteristics of the participated instructors (n = 100)

Age group	Frequencies	Percentage
20-30 years	4	4%
31-40 years	68	68%
41-50 years	28	28%
Specialty		
Community health	18	18%
Medical-surgical nursing	45	45%
Pediatric nursing	12	12%
Obstetrical & gynecological nursing	17	17%
Critical care nursing	8	8%
Nationality		
Saudi	30	30%
Egyptian	26	26%
Sudanese	22	22%
Jordanian	8	8%
Filipino	6	6%
Indian	4	4%
Yemeni	4	4%
Qualification		
Doctorate	95	95%
Master	4	4%
Bachelor	1	1%
Gender		
Male	34	34%
Female	66	66%
Years of teaching experience		
0-3	8	8%
4-7	32	32%
8-11	55	55%
More than 11	5	5%

**Figure 1:**
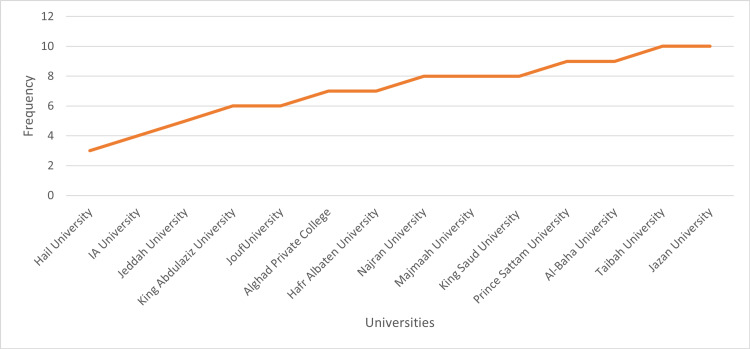
The universities of instructors (n = 100) IA: Imam Abdulrahman.

Figure 1 shows the universities of nursing instructors. They were from Jazan University (10%), Taibah University (10%), Albaha University (9%), Prince Sattam University (9%), King Saud University (8%), Majmaah University (8%), Najran University (8%), Hafar Al-Batin University (7%), Alghad Private College (7%), Jouf University (6%), King Abdulaziz University (6%), Jeddah University (5%), Imam Abdulrahman University (4%), and Hail University (3%).

Table 2 shows the nursing instructors' responses to technology and computer experiences for e-learning in Saudi Arabia. They can operate systems (4.47/5), navigate the internet (4.83/5), process Microsoft packages (4.73/5), prepare PowerPoint (4.81/5) and e-learning software courses (4.53/5), use video conference tools (4.31/5) and online chatting (4.42/5), and had the ability to teach through online platforms (4.36/5).

**Table 2:** Responses to study questions - technology and computer experience (n = 100) Min: minimum; Max: maximum; SD: standard deviation; #: the responses scored based on a five-point Likert scale.

Technology and computer experience	Min	Max	Mean^#^	SD
Operating systems (e.g., Windows XP, Windows, 7, Mac OSX, and Linux)	2	5	4.47	0.632
Internet (World Wide Web, WWW)	3	5	4.83	0.450
Word processing packages (e.g., Microsoft Word and WordPerfect)	2	5	4.73	0.524
Presentation software (e.g., PowerPoint)	1	5	4.81	0.539
Course management system for e-learning (e.g., WebCT, Blackboard, and Moodle)	1	5	4.53	0.721
Video-conferencing tools (e.g., Skype and Adobe Connect)	1	5	4.31	0.833
Online chat programs (e.g., Yahoo chat, AOL chat, and Facebook chat)	3	5	4.42	0.629
I have the necessary experience to teach via e-learning	3	5	4.36	0.606
Average instructors’ responses to technology and computer experience	2.13	5.00	4.55	0.46

Table 3 shows the nursing instructors' perception of e-learning in Saudi Arabian universities. They reported having required experiences (4.36/5), feeling confident to develop e-learning (4.45/5), and feeling confident to teach (4.52/5). The overall average rating of nursing instructors' perception of e-learning in Saudi universities was 4.56/5.

**Table 3:** Responses to study questions - instructors’ perception of e-learning (n = 100) Min: minimum; Max: maximum; SD: standard deviation; #: the responses scored based on a five-point Likert scale.

Variables	Min	Max	Mean^#^	SD
I have the necessary experience to teach via e-learning	3	5	4.36	0.606
I feel confident that I can develop an effective e-learning course	1	5	4.45	0.643
I feel confident that I can teach a successful e-learning course	2	5	4.52	0.656
1 feel confident using e-learning course management tools (e.g., Blackboard and WebCT)	2	5	4.47	0.633
I enjoy using computers in my teaching	2	5	4.78	0.513
I enjoy teaching e-learning courses	1	5	4.52	0.689
I enjoy developing e-learning courses	1	5	4.53	0.678
E-learning is an effective medium for learning	1	5	4.67	0.723
E-learning is an effective medium for learning	3	5	4.50	0.633
I can communicate efficiently through e-learning	1	5	4.61	0.691
I intend to teach e-learning courses when I am given the opportunity	1	5	4.63	0.688
I intend to use the Internet to support my teaching	1	5	4.59	0.651
I intend to use e-learning tools in my future teaching assignments	1	5	4.59	0.661
I am satisfied with using e-learning tools (e.g., Blackboard and WebCT) in my teaching	1	5	4.39	0.692
I am satisfied with developing my own e-learning courses	1	5	4.60	0.671
I am satisfied with using computers in my teaching	1	5	4.65	0.716
I like to use voice media instruction	1	5	4.56	0.729
I like to use image media instruction	2	5	4.65	0.639
I like to use animation media instruction	2	5	4.43	0.758
Average perception of instructors toward e-learning	2.11	5.00	4.5642	0.44407

Table 4 shows the correlation between the demographic characteristics (age, specialties, nationalities, qualifications, gender, and experiences) and the instructors’ technological experiences and perceptions. The one-way ANOVA test shows that technological and computer experience was significantly higher among nursing instructors aged 41-50 years compared to the other age groups (p = 0.01). It was also significantly higher among Indian instructors (4.87/5), followed by Saudi and Jordanian (4.64/5), and lowest among Egyptian instructors (4.12/5) with a p-value of 0.02. For the level of instructors' qualifications, doctorate instructors have higher experience (4.84/5), which is significantly higher than those who have master's or bachelor's degrees (p = 0.001). It shows that instructors' perceptions were significantly higher among instructors aged 41-50 years by 4.86/5 compared to the other age groups (p = 0.003). It was also significantly higher among Indian instructors (4.76/5) followed by Saudi (4.70/5) and Yemeni (4.65%) instructors, respectively, and lowest among Sudanese (4.18/5) with a p-value of 0.03. The level of instructors' qualification was another correlated factor; doctorate instructors have higher experience (4.76/5), which is significantly higher than those who have master's or bachelor's degrees (p = 0.002) by using the one-way ANOVA test.

**Table 4:** Comparison of demographic variables and the mean of instructors’ responses to technology and computer experience and instructors’ perception (n = 100) P: p-value; Freq.: frequency; SD: standard deviation; *: significant; #: the responses scored based on a five-point Likert scale.

Variables	Freq.	%	Technology and computer experience			Instructors’ perception of e-learning		
Age group			Mean	SD	p	Mean	SD	p
20-30 years	4	4%	3.10	1.20	0.01*	3.91	0.43	0.003*
31-40 years	68	68%	3.67	0.32		4.54	0.30	
41-50 years	28	28%	4.2	0.36		4.86	0.51	
Specialty								
Community health	18	18%	4.2	0.50	0.09	3.38	0.38	0.08
Medical-surgical nursing	45	45%	4.57	0.49		4.54	0.49	
Pediatric nursing	12	12%	3.83	0.41		4.23	0.77	
Obstetrical & gynecological nursing	17	17%	4.21	0.85		4.46	0.75	
Critical care nursing	8	8%						
Nationality								
Saudi	30	30%	4.64	0.61	0.02*	4.7	0.36	0.03*
Egyptian	26	26%	4.12	0.46		4.25	0.28	
Sudanese	22	22%	4.45	0.59		4.18	0.66	
Jordanian	8	8%	4.64	0.13		4.53	0.26	
Filipino	6	6%	4.38	0.16		4.52	0.17	
Indian	4	4%	4.87	0.16		4.76	0.23	
Yemeni	4	4%	4.54	0.61		4.72	0.96	
Qualification								
Doctorate	95	95%	4.84	0.37	0.001*	4.76	0.38	0.002*
Master	4	4%	4.55	0.91		4.23	0.83	
Bachelor	1	1%	3.89	0.99		3.94	0.43	
Gender								
Male	34	34%	4.65	0.50	0.09	4.84	0.48	0.6
Female	66	66%	4.72	0.35		4.81	0.49	
Years of teaching experience								
0-3	7	7%	4.12	0.89	0.03*	4.28	0.40	0.04*
4-7	21	21%	4.34	0.57		4.54	0.47	
8-11	68	68%	4.65	0.35		4.51	0.39	
More than 11	39	39%	4.94	0.42		4.64	0.51	

## Discussion

The current study was conducted among nursing instructors in Saudi universities, and they were from 14 different universities. The participants were from different nursing specialties and nationalities, including Saudi, Egyptian, Sudanese, Jordanian, Filipino, Indian, and Yemeni. They mainly have doctorate qualifications, and fewer have master’s or bachelor’s degrees in nursing. In contradicting our findings, one study conducted in Saudi Arabia on medical and health colleges indicated that most of the participants were female (66%), with variation in teaching experiences [16]. Similar findings were reported from one study on educators in Jordan, which showed that males are dominant among participants in the study [17]. Females were more predominant in this study because of the nature of the nursing profession and many Arabic countries did not graduate female nurses for years.

The nursing instructors’ experiences in technology and computer for e-learning was high. They can operate systems, navigate the internet, process Microsoft packages, prepare PowerPoint, have skills in video conference tools, and ability to teach through online platforms. One previous study on educators’ practices and attitudes toward e-learning in Jordan showed that the instructors may face barriers and challenges. The instructors reported that the areas of technology and computer skills are areas that required more enhancement and development, so more preparation and support are required before implementing e-learning [17]. In a separate online survey conducted by Eltaybani et al. in 2021, exploring the experiences of nursing students and educators toward e-learning during the pandemic, it was found that instructors had a significantly higher level of experience compared to students in utilizing e-learning techniques [18].

The study shows that technology and computer experience was significantly higher among nursing instructors aged between 41 and 50 years compared to other age groups, and among Indian, Saudi, and Jordanian instructors, and was lowest among Egyptians with a p-value of 0.02, and it was also higher among doctorate holders compared to master’s or bachelor’s degree holders (p = 0.001). This could be due to the nature of the university education they received in their countries.

The nursing instructors’ perception toward e-learning in Saudi Arabian universities was high; they feel confident to develop e-learning and teaching, and their overall perception of e-learning was very high. Similar findings from one study in Saudi Arabia in health and medical colleges indicated that the staff had positive attitudes toward e‑learning, especially after the pandemic [16]. According to Alanazi and Alshaalan (2020), a significant number of nursing instructors at Saudi universities showed confidence in developing e-learning courses and engaging in teaching activities using this method [16].

The findings of this study were higher than the level of perception reported in one study conducted by Alqabbani et al. in Saudi Arabia on readiness for e-learning during the pandemic, which indicated that about two-thirds of instructors at Princess Nourah Bint Abdulrahman University had a moderate level of perception on e-learning [19]. Other contradicting findings were reported from a study by Shambour et al. (2022) on perceptions of instructors on distance learning, which showed that no significant differences occurred during the pandemic [20].

The instructors' age, nationality, and qualification were influencing factors of instructors’ perception. It was found that instructors aged 41-50 years have a higher perception compared to the other age groups (p = 0.003), Indians followed by Saudi and Yemeni instructors have a higher perception of e-learning compared to others and the lowest level among Sudanese instructors (p = 0.03), and it was found that doctorate instructors have higher experience compared to instructors with master’s and bachelor’s degrees (p = 0.002). One previous study by Shambour et al. (2022) on the perceptions of instructors on distance learning showed that the level of qualification is an influencing factor in the instructors' perception while age is not significantly correlated with the level of perception [20].

There are some limitations of this study. It was conducted among a small sample size, which may limit the generalizability of the findings. Another limitation was the use of an online approach for data collection through which it was difficult to control bias.

## Conclusions

In conclusion, the study showed that the nursing instructors in Saudi universities have good experience in technology and computer and they also had a positive perception of e-learning during the pandemic. Instructors aged 41-50 years, Indian and Saudi instructors, and those who have doctorate qualifications were having significantly higher experience in technology and computer, with high levels of perception on e-learning during the COVID-19 pandemic. It is recommended to conduct further research on qualitative methods to deeply investigate the instructors’ views.
